# Towards a more accurate annotation of tyrosine-based site-specific recombinases in bacterial genomes

**DOI:** 10.1186/1759-8753-3-6

**Published:** 2012-04-13

**Authors:** Rob Van Houdt, Raphael Leplae, Gipsi Lima-Mendez, Max Mergeay, Ariane Toussaint

**Affiliations:** 1 Unit of Microbiology (MIC), Belgian Nuclear Research Centre, SCK•CEN, Boeretang 200 Mol B-2400, Belgium; 2 Department of Informatics, Campus du Solbosch - CP197, 50 avenue F.D. Roosevelt, Bruxelles 1050, Belgium; 3 Research Group of Bioinformatics and (Eco-)systems Biology, Department of Structural Biology, VIB, Pleinlaan 2, Brussels 1050, Belgium and Research Group of Bioinformatics and (Eco-)systems Biology, Microbiology Unit (MICR), Department of Applied Biological Sciences (DBIT), Vrije Universiteit Brussel, Pleinlaan 2, Brussels 1050, Belgium; 4 Laboratoire Bioinformatique des Génomes et Réseaux (BiGRe), Université Libre de Bruxelles, Bvd du Triomphe, Bruxelles 1050, Belgium

## Abstract

**Background:**

Tyrosine-based site-specific recombinases (TBSSRs) are DNA breaking-rejoining enzymes. In bacterial genomes, they play a major role in the comings and goings of mobile genetic elements (MGEs), such as temperate phage genomes, integrated conjugative elements (ICEs) or integron cassettes. TBSSRs are also involved in the segregation of plasmids and chromosomes, the resolution of plasmid dimers and of co-integrates resulting from the replicative transposition of transposons. With the aim of improving the annotation of TBSSR genes in genomic sequences and databases, which so far is far from robust, we built a set of over 1,300 TBSSR protein sequences tagged with their genome of origin. We organized them in families to investigate: i) whether TBSSRs tend to be more conserved within than between classes of MGE types and ii) whether the (sub)families may help in understanding more about the function of TBSSRs associated in tandem or trios on plasmids and chromosomes.

**Results:**

A total of 67% of the TBSSRs in our set are MGE type specific. We define a new class of actinobacterial transposons, related to Tn*554*, containing one abnormally long TBSSR and one of typical size, and we further characterize numerous TBSSRs trios present in plasmids and chromosomes of α- and β-proteobacteria.

**Conclusions:**

The simple *in silico *procedure described here, which uses a set of reference TBSSRs from defined MGE types, could contribute to greatly improve the annotation of tyrosine-based site-specific recombinases in plasmid, (pro)phage and other integrated MGE genomes. It also reveals TBSSRs families whose distribution among bacterial taxa suggests they mediate lateral gene transfer.

## Background

Tyrosine-based site-specific recombinases (TBSSRs) are well known DNA breaking-rejoining enzymes that belong to a superfamily that also includes type IB topoisomerases, including human topoisomerase I. The 3D structure and molecular mechanisms of action of several enzymes of the family are well documented [1-6].

TBSSRs are major actors in the roaming of mobile genetic elements (MGEs) in bacterial genomes. Very often called "phage-like integrases" because they were originally discovered on temperate phages (for example, λ, P2 and P22). TBSSRs do, however, i) occur on other types of MGEs and ii) catalyze various biological processes. These include the integration of temperate phage genomes to become prophages and of integrated conjugative elements (ICEs), their excision at the onset of lytic growth or conjugative transfer, the integration and excision of integron cassettes, the correct segregation of plasmids and chromosomes (reviewed in [7-9]) by resolution of dimers (or higher level multimers), the resolution of cointegrates resulting from the replicative transposition of some types of transposons [10], and the excision of specific DNA fragments responsible for the transient inactivation of genes (for a general review see [11]).

In the present genomic era, TBSSR annotation is far from homogenous, whether for genomes or in databases. Misinterpretation arises from the TBSSR property of catalyzing integration/excision reactions, which are also catalyzed by two other very different types of enzymes, the serine-based site specific recombinases (SBSSRs) and the DDE transposases, the latter being closely related to retroviral integrases, with which they share the conserved aspartate-glutamate-aspartate (DDE) catalytic residues.

Despite their abundance in prokaryotic genomes, including in plasmids where they appear as one of the largest conserved protein families ([12] and Additional file 1: Table S1), TBSSRs have not been so far extensively analyzed in terms of their relative sequence conservation among various types of MGEs or chromosomes. Boyd *et al. *[13] showed that TBSSRs encoded by genomic islands (GIs) inserted near a tRNA locus are phylogenetically closer than they are to phage encoded ones. Similarly, Ryan *et al. *[14] showed that Tn*4371*-like ICE TBSSRs are very similar and can be easily differentiated from phage ones. However, the sets of phage proteins used in those studies were small.

In this study, using a set of over 1,300 TBSSR protein sequences tagged with their genome of origin, we attempt to investigate: i) whether TBSSRs tend to be more conserved within than between classes of MGE types, that is, whether (sub)families of TBSSRs are specific to one (sub)type of MGE and ii) whether these (sub)families may help in understanding more about the function of the plasmid encoded TBSSRs. It is indeed striking that the sole *Cupriavidus eutrophus *H16 plasmid pHG1 is predicted to encode 22 TBSSRs of 280 or more amino acids (aa) http://aclame.ulb.ac.be/perl/Aclame/Genomes/prot_view.cgi?view=genome&id=mge:823). A rapid count of the number of TBSSRs in plasmids suggests that it far exceeds the number of proteins closely related to known plasmid dimer resolution enzymes (for example, Cre of prophage P1 [15]) or associated with integrons previously known in plasmids [16].

We carried out a clustering analysis of 1,309 TBSSRs encoded by plasmids, phages, predicted prophages and conjugative transposons (ICET_n4371 _[14,17]), Recombinases In Trio (RIT) and Bipartite Module (BIM) elements [18] and GIs. The protein sequences in each cluster/family were aligned to look for the presence of a possible catalytic domain. Each family was analyzed to determine whether TBSSR protein families were MGE type specific and to further investigate the plasmid encoded TBSSRs.

## Results

**Figure 1:**
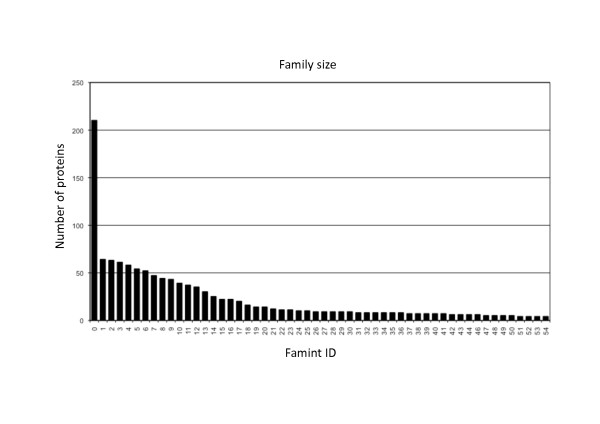
**Size distribution of TBSSR families**. Size distribution of the Famint families generated by MCL clustering at IF = 1.8 and E-value 0.01.

A set of 1,309 TBSSR protein sequences was assembled as described in Methods (Additional file 1: Table S2). Phage, plasmid and predicted prophage encoded proteins were retrieved from the ACLAME database, and GI, ICE_Tn*4371*_, RIT and BIM proteins from previously described sets of TBSSRs [13,18-20]. Far from being an exhaustive compilation of TBSSRs annotated in available sequenced genomes, this set has the advantage that each sequence can be easily traced to its associated genetic entity. Protein sequences were compared all vs. all and clustered using a combination of the SSEARCH and MCL algorithms (see Methods for details). This produced 102 families of TBSSR proteins, called Famint (for FAMily and INTegrase, Famint 0 to 44 and 46 to 102, Sup_Tables, Famint45 not being TBSSRs). Figure 1 summarizes the size and composition of the families consisting of 4 or more proteins (56 in total). Thirteen families with 3 proteins, 13 with 2 proteins and 21 singletons (including the conjugative transposon Tn*916 *TBSSR, 3 TBSSRs coded by phages/viruses, 8 by predicted prophages and 10 by plasmids) will not be considered further, unless they contain proteins associated with proteins in larger families.

It is readily apparent from Table 1 that there is a good overall separation between enzymes associated with various types of MGEs. Aside from a few exceptions, TBSSRs associated with chromosomal islands, plasmids or phage and prophages fall into distinct families. The TBSSR canonical catalytic motif is located in the C-terminal part of the protein and consists of a tyrosine residue (Y) separated by around 30 residues from an upstream arginine

**Table 1:** TBSSR family analysis

Famint ID	MGE type	No. prot	No. GI	No. phages	No. prophages	No. plasmids	Putative catalytic motif
0	intG	210	153	10(vir2)	38(pro2)	9(plas226)	no obvious one
1	RIT(A)	64^(4)^	ND	0	3	33(plas10)	RH-Y
2	RIT(C)	63^(4)^	ND	0	0	34(plas10)	RH-Y
3	mix	61	0	5(vir2)	15(fam202)	41(plas170)	RK-Y
4	mix	58	0	27(vir2)	29(pro2)	2(plas10)	RH-Y
5	RIT(B)	54^(4)^	ND	0	0	27(plas10)	RH-Y^(1)^
6	(pro)phage	52	0	16(vir2)	36(pro2)	0	RH/R-Y
7	(pro)phage	47	0	18(vir2)	29(pro2)	0	RHT/S-Y
8	IntI	44	0	0	1(pro2)	43(plas10)	RH-Y
9	plasmid	43	0	0	0	43(plas10)	RHS-Y^(1)^
10	plasmid	39	0	0	0	39(plas10)	RH-Y
11	plasmid	37	0	0	0	37(plas101)	R-Y
12	(pro)phage	35	0	8(vir2)	27(pro2)	0	RHT-Y
13	(pro)phage	30	0	11(vir2)	19(pro2)	0	RH-Y
14	Tn*4371 *^(4)^	25	13	0	5(pro2)	7(plas226)	RH-Y
15	(pro)phage	22	0	3(418)	19(pro76)	0	RSL or RLY-Y
16	(pro)phage	22	1	12(vir2)	9(pro2)	0	RHT-Y
17	(pro)phage	20	0	13(vir2)	7(pro2)	0	RHS-Y
18	plasmid	16	0	0	0	16(plas101)	RSG-Y
19	BIM(A)^(4)^	14	ND	0	0	8(plas10)	RH-Y
20	mix	14	0	1(vir2)	9 (pro2)	4(plas226)	R-Y
21	prophage, plasmid	12	0	0	3(pro2)	9(plas226)	RRT-Y
22	plasmid	11	0	0	0	11(plas10)	RRTF-Y
23	plasmid	11	0	0	0	11(plas10)	R-Y
24	prophage	10	0	0	10(pro2)	0	RK-Y
25	(pro)phage	10	0	4(vir2)	6(pro2)	0	RH-Y
26	(pro)phage	9	0	3(vir2)	6(pro2)	0	RH-Y
27	plasmid^(5)^	9	0	0	0	9(plas454)	RHT-Y^(2)^
28	phage, plasmid	9	0	0	4(pro2)	5(plas10)	RH-Y
29	mix	9	0	1(vir418)	7(pro76)	1(plas226)	R-Y
30	plasmid	9	0	0	0	9(plas688)	R-Y
31	plasmid	8	0	0	0	8(plas10)	RH-Y
32	plasmid	8	0	0	0	8(plas589)	R-Y
33	plasmid	8	0	0	0	8(plas10)	R-Y^(2)^
34	plasmid	8	0	0	0	8(plas10)	RAT-Y
35	(pro)phage	8	0	1(vir418)	7(pro76)	0	No R at expected distance from Y
36	plasmid	8	0	0	0	8(plas10)	RH-Y, partner 41, 90
37	mix	7	0	0	6(pro2)	1(plas10)	RH-Y
38	plasmid	7	0	0	0	7(plas101)	RR-Y
39	plasmid	7	0	0	0	7(plas589)	RH-Y^(3)^
40	plasmid	7	0	0	0	7(plas10)	RHS-Y
41	plasmid	7	0	0	0	7(plas454)	RR-Y
42	mix	6	0	0	1(pro2)	5(plas10)	RH-Y^(2)^
43	plasmid	6	0	0	0	6(plas10)	RR-Y^(2)^
44	plasmid	6	0	0	0	6(plas10)	RH-Y
46	plasmid	6	0	0	0	6(plas10)	RHT-Y
47	mix	5	0	3(vir2)	1(pro2)	1(plas226)	RH-Y
48	mix	5	0	3(vir2)	0	2(plas226)	R-Y
49	plasmid	5	0	0	0	5(plas10)	RRTAL-Y
50	(pro)phage	5	0	4(vir2)	1(pro2)	0	RHT-Y
51	(pro)phage	4	0	3(vir2)	1(pro2)	0	RHT-Y
52	prophage	4	0	0	4(pro76)	0	RK-Y(2)
53	plasmid^(5)^	4	0	0	0	4(plas454)	RH-Y, partner 62, one has no partner
54	plasmid	4	0	0	0	4(plas688)	RHTF-Y
55	plasmid	4	0	0	0	4(plas170)	R-Y
57-68		3					
69-81		2					
82-102		1					

The origins of the proteins can be traced by "vir", "plas" and "pro", which stand for ACLAME family IDs (vir2 is family:vir:2, plas10 is family:plasmids:10, pro2 is family: proph:2, and so on).

(pro)phage: the family contains proteins from both phages and prophages.

Only the most conserved R and adjacent amino-acids and the potential catalytic Y residue are mentioned.

(1) Not all proteins in the family have the conserved residues.

(2) A putative catalytic motif is discernible when two shorter sequences are removed from the alignment.

(3) Distance between RH and Y is around 50 residues.

(4) Some proteins in the family do not originate from ACLAME protein families, which is the reason why the number of GI, phages, prophages and plasmids do not add up to the number of proteins.

(5) Proteins in the family have a long N-terminal extension and are over 700 amino-acids long.

ND: Some proteins in the family could be part of a GI.

(R) followed by the residues required for the activation of the catalytic Y (for a review see [21]). Despite a variable degree of identity between the proteins within a family, the multiple alignments (accessible at http://aclame.ulb.ac.be/Resources/TBSSR/index.html) reveal a very well conserved (often 100% conservation) Y residue near the C-terminal end in almost all families, separated by around 30 residues from a conserved R (see Table 1), pointing towards the potential catalytic motif.

### Mixed families

The largest family, Famint0 (210 members), includes all but one of the GI proteins in the analyzed set. It also contains some proteins encoded by phages, predicted prophages and plasmids from the ACLAME family:vir:2, family:proph:2 and family:plasmids:226, respectively (see details in Table 1). Interestingly, TBSSRs from satellite phage P4, the so-called CP4-like islands [22] and phages F116, Sf6 and HK620 that have been reported to be similar to GI integrases [23] are part of this family. While all the GIs considered are located near a tRNA gene [13], this is the case for only 14 out of the 38 predicted prophages in this family (data not shown but accessible through http://aclame.ulb.ac.be/Tools/Prophinder/). Overall, proteins in Famint0 are not very well conserved. The family appears as a typical example of a large cluster generated by an automated procedure over a large dataset. Some sequences pull in relatively distantly related sequences, which in turn trigger the same effect, generating a pool of sequences most of which are related only through intermediates. This may be the reason for the absence of a recognizable conserved putative C-terminal catalytic tetrad in the multiple sequence alignment. Alternatively, GI enzymes may be non-functional due to a long-term selection for the preservation of the island.

Besides Famint0, only six other families (Famint3, 4, 14, 20, 28 and 48) are mixed and contain several proteins originating from at least two MGE types (plasmid, phage or prophage) (Table 1).

Famint3 is restricted to Firmicutes. The biological process performed by these plasmid proteins is hypothetical but since these plasmids are small, it could be the resolution of multimeric forms.

Famint14 contains TBSSRs encoded by a particular type of GIs, the conjugative transposons (or ICE) of the ICE*_Tn4371 _*family [14,17,20]. In the multiple alignment, they form a clear subgroup of very conserved sequences aside from plasmid and predicted prophage proteins, the latter of which do not appear as *bona fide *prophages (data not shown). In this family, no obvious closer relationship exists among proteins originating from more related hosts (data not shown).

Famint20 includes TBSSRs encoded by the shufflon elements present on conjugative plasmids R64 [24], R721 [25] and ColIb-P9 [26] and which, by inverting DNA segments, control the plasmids recipient specificity during mating in liquid media. The shufflon multiple inversion system consists of the TBSSR coding gene and several invertible DNA segments containing partial *pilV *genes separated by recombination sites. Recombination between any two inverted sites promotes the inversion of DNA segments independently or in groups, leading to the construction of several *pilV *genes with a constant N-terminal but different C-terminal segments. The resulting PilV products are adhesins located at the tip of the plasmid encoded type IV pilus, which recognizes lipopolysaccharides on the recipient cell (plasmid 153 kB from *Yersinia pseudotuberculosis *IP 31758 has a single *pilV *gene next to the TBSSR gene). None of the nine predicted prophages contributing to Famint20 bears or flanks a shufflon-like structure. Instead, they contain genuine phage-like genes and their TBSSR, despite being in most cases annotated "shufflon-specific DNA recombinase", appears to belong to a full or incomplete prophage. In addition, while the plasmids contributing to the family are hosted by γ-proteobacteria, the predicted prophages are in β-proteobacteria.

Famint28 includes proteins from plasmids and low score predicted prophages with no genuine phage characteristics besides replication. Only one *Desulfovibrio desulfuricans *predicted prophage has all expected features for being a functional prophage.

Overall, 400 proteins, that is, 30% proteins in the set do not group into MGE specific families.

### Plasmid resolvases?

The P1 Cre resolvase, a TBSSR expressed by the *E. coli *P1 circular plasmid prophage, is among the best structurally and biochemically characterized TBSSRs [27]. Upon clustering of phage, predicted prophage and plasmid proteins in ACLAME (version 0.4), P1 Cre joins with plasmid proteins in family:plasmids:101, pointing to the possibility that these proteins are plasmid dimer resolution enzymes. However, in the present analysis, P1 Cre belongs to a small family of only three proteins (Famint58), making this assumption shaky. ACLAME family:plasmids:101 splits here into Famint11 (which contains 37 plasmid proteins, 5 with less than 200 aa and, hence, most likely defective, and 32 of over 300 aa) and Famint18 with 16 plasmid proteins. Famint11 proteins belong to plasmids from very different hosts and several contribute two proteins to the family. Proteins in the pairs are not identical but more closely related than they are to the rest of the family members (data not shown). Famint18 contains proteins from plasmids residing in plant-interacting bacteria (except for *Nitrobacter hamburgensis *X14 plasmid 2). One pSymA plasmid contributes two proteins to the family. Putative catalytic sites derived from multiple alignments of Famint11, 18 and 58 members, respectively, are not the same. The present analysis thus brings no further support to a plasmid resolution function.

### Integrons

Integron-encoded integrases IntI are in Famint8. The 25 IntI proteins, associated with one to eight cassettes, are almost identical at the nucleotide level. Almost all of them have been described earlier (see the plasmid names and hosts in Additional file 1: Table S1). Integrons are often associated with IS elements or transposons (Tn) that ensure their horizontal spreading (see [16]). Hence, we expected at least some IS and Tn to tend to remain associated on different plasmids. This can be readily evaluated using pre-compiled Evolutionary Conserved Modules (ECM), that is, sets of genes with similar phylogenetic profiles [28] available in the ACLAME database for different similarity thresholds (sig). IntI proteins belong to the ACLAME family:plasmids:10, which is part of ECM9, sig10. ECM9 includes, among other protein families, Tn*3*-like transposases, SSSRs (resolvases) and IS6 transposases. This reflects the frequent association of integrons with either Tn*21*-like (Tn*3*-related) transposons, which encode these two types of proteins [29], or composite transposons, including two copies of IS*6 *(Tn*1548 *in pCTX_M3 and others) [30]. This grouping most likely results from the huge selective pressure imposed on bacterial populations by the overuse and release of antibiotics. It will be interesting to see whether these associations remain significant when more plasmid sequences of more various origins will be available. The association of integrons with Tn*402 *and related transposons [31], typified by the presence of the *tniA-tniB *and sometimes *tniQ *genes, appears weaker since these genes are not in ECM9 but form ECM45, with mercury resistance genes (although these also occur in integrons of Tn*3*-related transposons). Most other integron cassettes are in ECM13, reflecting their tendency to remain associated. Together ECM9 and 13 support the association of integrons with transposons and cohesion of the integron cassettes.

### BIM elements

Famint19 regroups nine TBSSRs from β-proteobacterial hosts. Members that were originally pointed out during the annotation of the *C. metallidurans *CH34 genome are associated with a second conserved protein of unknown function (Famint45) making up the bipartite module [18]. The NCBI Protein Clusters were used to have a more complete view of these two-genes associations (Additional file 1: Table S3); however, the number of strains harboring these modules remains too low to draw any conclusion about the exact nature of this association.

### TBSSR combinations

#### Tn554-related TBSSRs

The ACLAME family:plasmids:454 contains 20 abnormally long TBSSRs of 611 to 828 aa. Most originate from plasmids hosted by Actinobacteria. With the clustering procedure used here, the 20 proteins split into three smaller families of 9 (Famint27), 7 (Famint41) and 4 (Famint53) members, respectively. Most of these are associated with a second, adjacent and shorter TBSSR (around 350 aa) originally in the ACLAME family:plasmids:10 and here in Famint33 (partner of Famint27), Famint36 (partner of Famint41) and Famint62 (partner of Famint53). In one case, the two partners belong to Famint53 and 33, respectively. This couple resides on a *Bacillus cereus *plasmid and it is the only case, together with the α-proteobacterium *Novosphingobium aromaticivorans*, where the host is not an Actinobacterium.

The genes corresponding to most of the couples whose members belong to Famint27 and 33 and Famint53 and 62, are transcribed in the same direction and are associated with a third gene/protein, also similarly oriented. These third partners are found in ACLAME family:plasmids:1417 and are related to the TnpC protein of Tn*554 *from *Staphylococcus aureus *[32]. Consistent with this, Famint33, 36 and 62 proteins share significant similarity with Tn*554 *TnpB and Famint27 and Famint53 partners with Tn*554 *TnpA (data not shown). The Famint41 proteins are less related to Tn*554 *TnpB and have no obvious TnpC partner. Sets of contiguous genes corresponding to proteins in the same family align at the nucleotide level and these sequences can also be found in chromosomes of other Actinobacteria (*Mycobacterium, Streptomyces, Rhodococcus*, Table 2). The NCBI Protein Clusters provide a direct view of these sets of contiguous related clusters, which fit well with the Famint for the genomes common to the two data sets (Table 2).

**Table 2:** Tn*554*-like elements in plasmids and chromosomes

Genbank **Acc. No**.	Plasmids	**sTBSSR CLSK No**.	TnpB ID	**LTBSSR CLSK No**.	TnpA ID	TnpC
NC_008270	*Rhodococcus *sp. RHA1 pRHL2	636892	36	636891	41	none
NC_008697	*Nocardioides *sp. JS614 pNOCA01	636892	36	636891	41	none
NC_008537	*Arthrobacte*r sp. FB24 plasmid 1	636892	36	636891	41	none
NC_003903	*Streptomyces coelicolor *pSCP1 (2 copies)	636892	36	636891	41	none
NC_007491	*Rhodococcus erythropolis *PR4 pREL1	636892	36	636891	41	none
NP_898763	*R. erythropolis *pBD2	636892	36	636891	41	none
NC_008271	*Rhodococcus *sp. RHA1 pRHL3	523288	33	2316403	27	2316404
NC_007491	*R. erythropolis *PR4 pREL1	2526550	33	none	27	2526549
NP_898763	*R. erythropolis *pBD2	647580	33	647579	27	647578
NP_898763	*R. erythropolis *pBD2	826662	33	none	27	647587
NC_005707	*Bacillus cereus *pBc10987	no cluster	33	2462320	53	925958
NC_009426	*Novosphingobium aromaticivorans *DSM 12444 pNL1	782394	62	782395	53	782393
NC_009427	*N. aromaticivorans *DSM 12444 pNL2 (2 copies)	782394	62	782395	53	782393
NC_009339	*Mycobacterium gilvum *PYR-GCK pMFLV01	no cluster	33	no cluster	27	no cluster
NC_009339	*M. gilvum *PYR-GCK pMFLV01	647579	33	730723	27	647580
NC_008147	*Mycobacterium *sp. MCS plasmid 1	776375	33	776376	27	772808
NC_008704	*Mycobacterium *sp. KMS pMKMS02	776375	33	776376	27	772808
NC_004719	*Streptomyces avermitilis *MA-4680 SAP1*	no cluster	33	none	_	none

**Genbank Acc. No**.	**Chromosomes**	**sTBSSR CLSK No**.	**TnpB ID**	**LTBSSR CLSK No**.	**TnpA ID**	**TnpC**

NC_008596	*Mycobacterium smegmatis *str. MC2 155	776375	_	776376	_	772808
NC_008711	*Arthrobacter aurescens *TC1	776375	_	776376	_	772808
NC_012803	*Micrococcus luteus *NCTC 2665 (2 copies)	776375	_	776376	_	772808
NC_008705	*Mycobacterium *sp. KMS	776375	_	776376	_	772808
NC_008726	*Mycobacterium vanbaalenii PYR-1*	776375	_	776376	_	772808
NC_013235	*Nakamurella multipartita *DSM 44233 (7 copies)	647580	_	647579	_	647578
NC_010397	*Mycobacterium abscessus *ATCC 19977 (2 copies)	647580	_	647579	_	730718
NC_009338	*Mycobacterium gilvum *PYR-GCK	647580	_	647579	_	647578
NC_009077	*Mycobacterium *sp. JLS	647580	_	647579	_	730718
NC_008726	*M. vanbaalenii PYR-1 *(4 copies)	647580	_	647579	_	647578
NC_008726	*M. vanbaalenii PYR-1*	647580	_	647579	_	
NC_008726	*M. vanbaalenii PYR-1*	647580	_	647579	_	none
NC_008595	*Mycobacterium avium *104	647580	_	647579	_	none
NC_008268	*Rhodococcus jostii *RHA1	647580	_	647579	_	none

CLSK No., Protein Cluster Database Number; ID, Famint number; none, there is no equivalent annotated protein at that position. no cluster, the protein is annotated but not part of a cluster. *, plasmid SAP1 has a truncated version of the Famint33 protein (246 aa only) and has no partners associated. -, not in the set of analyzed TBSSR proteins. s, shorter, L, longer.

Tn*554 *has a unique integration site [33]. Some of the genomes that carry the elements discussed here have two or more identical copies of the same *tnpAB(C) *association (for example, *Mycobacterium vanbaalenii *PYR-1 chromosome, *Streptomyces coelicolor *pSCP1 plasmid). They could have several identical or very similar *attB *sites as well, especially when the two copies are on the chromosome and a plasmid (*Mycobacterium *sp. MCS chromosome and pMKMS02 plasmid). Some plasmids also have copies of different variants (pREL1 from *Rhodococcus erythropolis *PR4, pBD2 from *R. erythropolis *BD2; Table 2). At least some of these elements ought to be mobile since identical copies are found on chromosomes and plasmids and on different plasmids (identical copies at the nucleotide level in *Mycobacterium *sp. MCS chromosome and pMKMS02 plasmid, and pNL1 and pNL2 plasmids, respectively; data not shown).

Tn*554 *TnpC stimulates transposition and influences the orientation of transposed copies [34]. It may thus be dispensable, which could explain its absence from some of the related elements. Alternatively, unrelated proteins could be TnpC homologues although inspection of TnpB neighbors does not support this view.

#### RIT elements: TBSSRs in trio

Famints1, 2 and 5 contain proteins that are encoded by three adjacent and overlapping genes, *ritA, ritB *and *ritC*. These TBSSR trios were first described in *C. metallidurans *CH34 [18]. Although not particularly well conserved, the three proteins in RITs make distinct families (RitA in Famint1, RitB in Famint5 and RitC in Famint2). They are particularly abundant in plasmid pHG1 from *C. eutrophus *H16. As shown in Table 1, all three families display a possible catalytic motif, suggesting that the three enzymes may be active, although it is still difficult to understand how a combination of three proteins would be needed to cleave four DNA strands in a breaking and rejoining reaction.

To access a larger and precompiled set of RIT TBSSRs, we again used the NCBI Protein Clusters, (Table 3 and Additional file 1: Table S4). As expected from the method used to assemble them, which is more stringent than our clustering procedure, these clusters are more granular, but nevertheless still clearly separate the A, B and C types of RIT encoded enzymes. To a few exceptions, these remain associated in trios of distinct clusters, with characteristic short overlaps between open reading frames (four to eight base pairs). Apparently, RITs are more frequent in chromosomes (in 62.3% of the cases) than in plasmids (in 37.7% of the cases). For 19 chromosomally-embedded RITs more information is available on the genomic context (through literature and Islandviewer [35]), indicating that for this group approximately 68% is located on a predicted genomic island.

**Table 3:** RIT elements classified according to NCBI Protein Clusters

RIT type	RitA Famint1	RitB Famint5	RitC Famint2
RIT1	CLSK521097	CLSK2306416	CLSK2306415
RIT2	CLSK2407259	CLSK2314503	CLSK458345
RIT3A	CLSK382373	CLSK747445	CLSK502077
RIT3B	CLSK2525360	CLSK747445	CLSK502077
RIT3C	CLSK382373	CLSK747445	CLSK739704
RIT4A	CLSK516123	CLSK809014	CLSK893868
RIT4B	CLSK778800	CLSK809014	CLSK893868
RIT5A	CLSK891968	CLSK891969	CLSK891970
RIT5B	CLSK954479	CLSK2468126	CLSK891970
RIT6	CLSK953941	CLSK953942	CLSK953943
RIT7A	CLSK2477616	CLSK923804	CLSK2477617
RIT7B	CLSK962675	CLSK923804	CLSK2332435
RIT7C	CLSK2809109	CLSK923804	CLSK2809110
RIT8	CLSK971115	CLSK971116	CLSK971117
RIT9	CLSK502016	CLSK537353	CLSK864421
RIT10	CLSK2491249	CLSK2321302	CLSK923805
RIT11	CLSK2471156	CLSK962653	CLSK962652

RITs with the same number include at least one common protein cluster.

In the absence of experimental results related to the mobility of the RIT structures, their distribution among different taxa and multiple copies in a genome provide some hints into this question. In particular, RITs with RitB in cluster CLSK923804 (group RIT7), which is associated with several types of RitA and RitC, are present in Firmicutes, α-, β- and δ-proteobacteria (Additional file 1: Table S4). Identical RIT copies are found in *Burkholderia phytofirmans *PsJN (three copies), *Aromatoleum aromaticum *EbN1 (three copies), *Dinoroseobacter shibae *DFL 12 (two copies), *Heliobacterium modesticaldum *Ice1 (two copies), *Bordetella petrii *DSM 12804 (two copies), *Caulobacter *sp. K31 (three copies), *Mesorhizobium loti *MAFF303099 (two copies) and *Gramella forsetii *KT0803 (two copies).

The RIT elements present in two strains of *Acidithiobacillus ferrooxidans *(ATCC 23720 and 53993), which are all from the same type (Additional file 1: Table S4 and Additional file 1: Table S5), are located in the transposase gene of a transposon related to Tn*6049 *from *C. metallidurans *CH34. This particular insertion site supports the mobility of this RIT, which is, however, tempered by the fact that these composite Tn::RIT structures are almost identical at the nucleotide sequence level and inserted at the same genomic location. The presence of a RIT6 insertion in the *radC *gene of Tn*6054 *(data not shown), a Tn*4371*-like ICE of *C. metallidurans *CH34 [17,18,20] also supports RIT mobility. Finally, on some plasmids (especially pHG1 from *C. eutrophus *and plasmid 2 from *A. aromaticum *sp. EbN1, Additional file 1: Table S4), RITs appear in complex combinations, where one or more of the RIT CDS is missing or truncated, again pointing towards some "mobility/recombination" activity.

Since some strains contain two or more copies of the same RIT element, it was possible to deduce the length of the RIT to be around 3,500 bp. However, there seems to be some sequence variation at the ends of the element. Search for direct and inverted repeats in the sequence as potential TBSSR binding sites did not produce any convincing result besides a 28-9 bp inverted repeat and overlapping palindromic sequence flanking *ritA *and *ritC*, respectively. These features do not fit well the usual multiple core and arm TBSSR binding sites found on, for example, temperate phage and ICE genomes.

#### Distribution of TBSSRs families among bacterial species and genera

**Figure 2:**
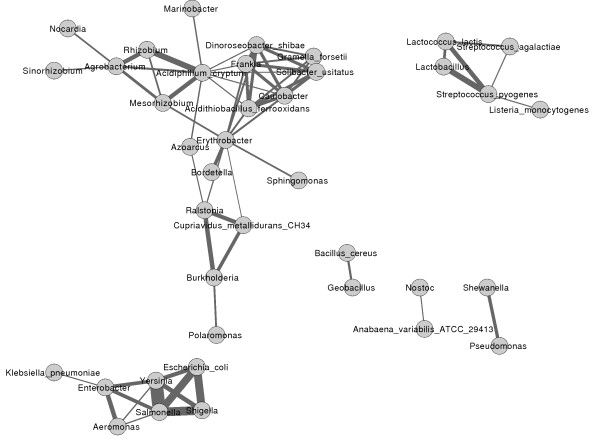
**Weighted graphical representation of Famint families shared between bacterial hosts**. Bacterial strains were grouped at the genus level unless there was a single representative at the strain or the species level. These groups of bacteria were represented in terms of the Famint families they contain. The graph was built as described in Methods. Nodes are bacterial genera, species or strains. They are linked by an edge if sharing Famint families. The thickness of the edges is proportional to the number of families shared by linked nodes. Note the tight grouping of Enterobacteria and Firmicutes.

To investigate the network of associations between bacterial groups given by the presence of different Famints, we linked pairs of the bacterial hosts based on the number of Famints they share (see Methods for details). The network (Figure 2) consists of six disjoint components. Five of these components are small and phylogenetically homogeneous. Two of these five harbor, respectively, five Firmicutes hosts and seven γ-proteobacteria, all of which are Enterobacteria. Interestingly, the latter separate from the rest of the proteobacterial hosts, which are found in the largest component. This may reflect the specialized habitat of Enterobacteria. In contrast, the largest component embraces hosts from different phyla, Proteobacteria, Actinobacteria, Acidobacteria and Bacteroidetes Two hubs mediate the inter-phyla and inter-class links, thus holding the component together. *Erythrobacter *and *Acidiphilum *connect α with β- and γ-proteobacteria, and with Actinobacteria and Acidobacteria. Should these two hubs be removed, the component would split into two singletons and three clusters, two of which respectively include α- and γ-Proteobacteria. *Nocardia*, an Actinobacteria, makes a single connection with *Agrobacterium *in the α-proteobacteria subgroup. The third cluster displays a heavy connectivity (number and weight of the links) and is phylogenetically very heterogeneous. It is composed of *Frankia *(Actinobacteria), *Dinoroseobacter *and *Caulobacter *(γ-proteobacteria), *Acidithiobacillus *(γ-proteobacteria), *Gramella *(Bacteroidetes), *Solibacter *(Acidobacteria). This network has to be taken cautiously, since the bias in the types of MGEs represented in the various hosts considered in our study cannot be neglected.

## Discussion

### The majority of tyrosine-based site-specific recombinases are specific to the type of mobile genetic element

MCL clustering organized 27% of the 1,309 TBSSR proteins encoded in plasmids, (pro)phages, GIs and ICEs into five "mixed" families, including the largest Famint0 of 210 members (that is, 16%) that comprises all but one IntG proteins from GIs. Thus two-thirds of the TBSSR set (63%) fit into families of four or more members that belong to the same type of MGE. Most families display a conserved potential catalytic motif (Table 1). Thus, by providing hints into the nature of the MGE type containing the protein, which can be further assessed by the function of neighboring genes on the genome of origin, the simple procedure used here could contribute greatly to improve the annotation of tyrosine-based site-specific recombinases.

A large proportion of the TBSSRs belonging to plasmids can still not be associated with any biological process. In particular, we could not unravel the families that promote the resolution of co-integrate and/or plasmid di-multi-mers. We did, however, identify families associated with new types of possible mobile elements.

First, a family of *S. aureus *Tn*554*-related transposons is present in Actinobacteria, with two adjacent TBSSRs coding genes. One of the proteins has a long N-terminal extension (over 400 aa) that displays no similarity with any obvious recognizable conserved protein domain and contains a potential C-terminal catalytic motif. Such a motif is also found on the shorter TBSSR in the tandems. In Tn*554 *both proteins are required for transposition [34], although their exact role in the reaction has not been established.

Second, the BIM associated TBSSR were so far found in a limited range of hosts all in the β-proteobacteria class, such that the mobility of BIMs presently lacks even theoretical support.

Third, the RIT elements are made of TBSSR trios and predominate in β- and α-proteobacteria. The distribution of different sub-types of RITs among these bacterial genera supports the hypothesis of their mobility, which nevertheless remains to be experimentally demonstrated.

### Improving the annotation of site specific and transpositional recombinases

A more robust annotation of site specific and transpositional recombinases is desirable to avoid the propagation of, for example, 'phage-like integrase' annotation for a Tn*4371*-like ICE encoded TBSSR. We show above that with a limited set of TBSSRs of known origin, it becomes possible to infer the type of MGE coding for the enzyme in two-thirds of the cases. However, specifying the nature of the coding MGE is not sufficient to design a robust annotation of the protein. One way to go is to use a structured ontology, such as the GO ontology (http://www.geneontology.org), which already offers a number of options that could be expanded. All TBSSRs belong to the same category of enzymes (molecular function in GO terminology), that is, "tyrosine-based site-specific recombinase activity" (GO:0009037) and catalyze the same biological processes "DNA integration" (GO:0015074) and "DNA excision" (GO:0044349) and should be annotated as such. When supported by the function of neighboring genes, the nature of the associated MGE sequence could be specified (using, for instance, the SO sequence ontology; http://www.sequenceontology.org/). In cases where the nature of the sequence provides a hint into a more precise biological process catalyzed by the TBSSR, these could as well be specified as "establishment as a prophage" or, "establishment as a GI", "resolution of plasmid dimers" or "resolution of cointegrates", using again defined terms as those offered by GO (or to be so but already available in the dedicated MeGO ontology http://aclame.ulb.ac.be/Classification/mego.html).

As outlined in the introduction, TBSSRs catalyze the same biological processes as SBSSRs and DDE transposases/integrases, which also suffer from the absence of a coherent annotation. A similar approach to better discriminate these two other categories of proteins could be envisaged. The much higher sequence conservation of the SBSSRs may be a limitation, but there, enzymes that catalyze integration/excision reactions appear much larger (around 500 aa, [36]) than those that catalyze inversion reactions (200 aa). In the case of DDE transposases/integrases, assembling a coherent set of sequences with indisputable MGE type origin is not straightforward, except for DDE enzymes encoded by IS sequences that are robustly classified in families available in the IS-Finder database (http://www-is.biotoul.fr/is.html, [37]). Nevertheless, considering separately the molecular function and the biological processes resulting from these enzyme activities would here again contribute to a more informative sequence annotation as in the case of TBSSRs.

## Conclusions

A simple *in silico *procedure that uses a set of reference TBSSRs from defined MGE types combined with the analysis of the genetic context would allow for a greatly improved annotation of up to 60% of the tyrosine-based site-specific recombinases in prokaryotic genomes. In addition, the distribution among bacterial taxa of TBSSRs families should help to identify new types of mobile genetic entities deserving further experimental characterization.

## Methods

A set of 1,309 TBSSR sequences (Additional file 1: Table S2) was assembled by grouping: i) 154 GI encoded proteins described by [13], ii) the proteins in all plasmid (1,109), phage (457) and predicted prophage (760) protein families annotated as TBSSR (GO:0009037) in ACLAME DB version 0.4 [19], iii) a manually compiled list of proteins encoded by Tn*4371*-like transposons [20] and iv) proteins from RIT and BIM elements. The latter were identified by synteny using *C. metallidurans *genome sequence [18] on the MAGE annotation package [38] and the NCBI Protein Clusters [39]. A small number of identical proteins present in the ACLAME and additional lists, which are readily visible in the multiple alignments, were not sorted out. Protein sequences were clustered using the SSEARCH-MCL algorithm combination as follows: 1) Each sequence was used as a query sequence for SSEARCH to scan the set itself. The hits were limited to an E-value of 0.01; 2) all the query-hit pairs + the log10 (E-value) obtained with SSEARCH were collected and provided as an input similarity matrix to the MCL algorithm; 3) MCL was executed with inflation factors (parameter influencing the clusters granularity) ranging from 1.2 (minimal value) up to 8.0 (maximum value) by steps of 0.2, each giving a different sets of clusters. To select the optimal clustering, the homogeneity of the sets was assessed by calculating the intra-cluster clustering coefficient (ICCC, see [28] for the detailed procedure). Briefly, the ICCC measures the degree of inter-connectivity, as defined in the similarity matrix, of proteins within the same cluster. The inflation factor 1.8 gave the highest ICCC and was, therefore, selected as the optimal clustering result referred in this article as the protein families or Famints. The sequences from each cluster were provided to MUSCLE [40] using the default parameters to build the multiple sequence alignments. Each family was analyzed manually. Multiple alignments were visualized with the Jalview display and calculation package [41]. Putative catalytic sites were determined by visual inspection of the multiple alignments. Associations of protein families in ECMs [28] were retrieved from ACLAME version 0.4. Sharing of Famints families by hosts was analyzed using the Compare classes/clusters and Convert Graph methods provided on the NeAT web interface (http://rsat.bigre.ulb.ac.be/rsat/index_neat.html) [42], and the Cytoscape graph display [43].

## Abbreviations

aa: amino acid; BIM: bipartite module; ECM: Evolutionary Conserved Modules; GI(s): genomic island(s); ICCC: intra-cluster clustering coefficient; ICEs: integrated conjugative elements; MGE(s): mobile genetic element(s); MUSCLE: multiple sequence comparison by log-expectation; RIT: recombinase in trio; SBSSR(s): serine-based site specific recombinase(s); TBSSR(s): tyrosine-based site-specific recombinase(s)

## Competing interests

The authors declare that they have no competing interests.

## Authors' contributions

RVH and MM made the RIT and BIM analysis. RL maintains the ACLAME database and performed the computer procedures in this work. AT analyzed the Famint clusters, multiple alignments and NCBI Protein Clusters. GLM computed the graph in Figure 2. All the authors contributed to writing the manuscript.

## Supplementary Material

Additional file 1 **Spreadsheet containing the supplementary data in the different sheets**. Sheet TBSSR_plasmid_S1_ **Table S1**. Number of TBSSR genes in plasmid genomes in ACLAME version 0.4. BP: base pairs. Sheet Famints_S2 **Table S2**. TBSSR family (Famint) composition. Sheet BIM_ S3 **Table S3**. Identified BIM elements and associated NCBI Protein Clusters. SMC domain protein: predicted function of the second protein encoded by the element. Sheet RIT_S4 **Table S4**. Distribution of various RIT types among bacterial strains and composition of RIT types in terms of NCBI Protein Clusters. CDS distance is the distance in base pairs between the RitA and RitB **(A-B) **and RitB and RitC **(B-C)**. Sheet Tn6049_RIT_S5 **Table S5**. Tn*6049 *related transposons in terms of NCBI Protein Cluster composition, some of which with an insertion of a RIT2 element. (XLS 256 kb). Click here for file
